# *IKBKB* siRNA-Encapsulated Poly (Lactic-*co*-Glycolic Acid) Nanoparticles Diminish Neuropathic Pain by Inhibiting Microglial Activation

**DOI:** 10.3390/ijms22115657

**Published:** 2021-05-26

**Authors:** Seounghun Lee, Hyo-Jung Shin, Chan Noh, Song-I Kim, Young-Kwon Ko, Sun-Yeul Lee, Chaeseong Lim, Boohwi Hong, Sin-Young Yang, Dong-Woon Kim, Won-Hyung Lee, Yoon-Hee Kim

**Affiliations:** 1 Department of Anesthesiology and Pain Medicine, Chungnam National University Sejong Hospital, 20 Bodeum 7-ro, Sejong 30099, Korea; anelee1982@cnuh.co.kr; 2 Department of Anesthesiology and Pain Medicine, College of Medicine, Chungnam National University, 282 Munhwa-ro, Jung-gu, Daejeon 35015, Korea; jyfchrh@cnuh.co.kr (C.N.); annn8432@gmail.com (Y.-K.K.); neoquack@gmail.com (S.-Y.L.); limtwo2@gmail.com (C.L.); koho0127@gmail.com (B.H.); 3 Department of Anatomy and Cell Biology, Chungnam National University College of Medicine, Daejeon 35015, Korea; shinhyo1013@gmail.com (H.-J.S.); kthddl2295@gmail.com (S.-I.K.); visnu528@gmail.com (D.-W.K.); 4 Brain Research Institute, School of Medicine, Chungnam National University College of Medicine, Daejeon 35015, Korea; 5 Department of Anesthesiology and Pain Medicine, Chungnam National University Hospital, 282 Munhwa-ro, Jung-gu, Daejeon 35015, Korea; 6 Department of Medical Science, Chungnam National University College of Medicine, Daejeon 35015, Korea; 7 Department of Anesthesiology and Pain Medicine, Daejeon Veterans Hospital, 147 Daechoong-ro 82, Daedeok-gu, Daejeon 34314, Korea; y3name@naver.com

**Keywords:** neuropathic pain, microglia, *IKBKB*, siRNA, poly (lactic-*co*-glycolic acid) nanoparticle

## Abstract

Activation of nuclear factor-kappa B (NF-κB) in microglia plays a decisive role in the progress of neuropathic pain, and the inhibitor of kappa B (IκB) is a protein that blocks the activation of NF-κB and is degraded by the inhibitor of NF-κB kinase subunit beta (IKBKB). The role of *IKBKB* is to break down IκB, which blocks the activity of NF-kB. Therefore, it prevents the activity of NK-kB. This study investigated whether neuropathic pain can be reduced in spinal nerve ligation (SNL) rats by reducing the activity of microglia by delivering *IKBKB* small interfering RNA (siRNA)-encapsulated poly (lactic-*co*-glycolic acid) (PLGA) nanoparticles. PLGA nanoparticles, as a carrier for the delivery of *IKBKB* genes silencer, were used because they have shown potential to enhance microglial targeting. SNL rats were injected with *IKBKB* siRNA-encapsulated PLGA nanoparticles intrathecally for behavioral tests on pain response. *IKBKB* siRNA was delivered for suppressing the expression of *IKBKB*. In rats injected with *IKBKB* siRNA-encapsulated PLGA nanoparticles, allodynia caused by mechanical stimulation was reduced, and the secretion of pro-inflammatory mediators due to NF-κB was reduced. Delivering *IKBKB* siRNA through PLGA nanoparticles can effectively control the inflammatory response and is worth studying as a treatment for neuropathic pain.

## 1. Introduction

Neuropathic pain is caused by tissue or nerve damage, which may result in chronic pain, such as spontaneous pain, allodynia, or hyperalgesia [1]. Diabetic neuropathy, post-herpetic neuralgia, trigeminal neuralgia, and cancer pain have causes other than simple nerve damage or trauma [2]. These pains often do not respond to general nonsteroidal anti-inflammatory drugs (NSAIDs) or opioids. Tricyclic antidepressants, pregabalin, and gabapentin, which are commonly used medications, do not provide adequate treatment owing to a lack of understanding of the mechanisms of neuropathic pain generation and development. Therefore, to effectively treat chronic neuropathic pain, it is important to investigate its mechanisms. Recent studies have shown that microglia play an important role in initiating, maintaining, and mediating neuropathic pain.

Microglia are quiescent immune cells of the central nervous system [3]. When microglial cells are activated, they can act as phagocytes [4], present antigens to T lymphocytes, and release mediators containing proinflammatory cytokines [5]. Activation of microglia is characterized by the expression of several markers, such as activation of type 3 complement receptors, ionized calcium-binding adapter molecule 1 (Iba1), cluster determinant 14, toll-like receptor 4 [6], and p38-mitogen activated protein kinase [7]. In neuropathic pain, microglia are one of the first spinal cord cell types to be activated within 4 h after peripheral nerve injury [8]. Several studies have confirmed the weakening of pain hypersensitivity by blocking microglial activation [9,10,11].

Nuclear factor-kappa B (NF-κB) is a transcription factor that plays a critical role in cell survival and immune response. As with many other factors, NF-κB functions differently depending on the type of cells and the time of action. Increased NF-κB activity in immune and nervous system cells is associated with inflammation- and nerve damage-induced pain in animals as well as several chronic pains in humans [12]. NF-κB is also involved in the activation of microglia and plays a central role in the production of reactive oxygen species and proinflammatory cytokines (IL-1β, interferon-γ, tumor necrosis factor alpha [TNF-α], etc.) that can induce secondary neurotoxicity [13,14].

Recent studies have shown that small-interfering RNA (siRNA), also called silencing RNA, interferes with the expression of certain genes by breaking down mRNA after transcription, therefore preventing translation [15]. siRNA can be used for the inhibition of genes that act as mediators of disease pathology. However, there are limitations to the use of siRNAs. The strong anionic charge of the phosphate backbone prevents effective diffusion due to electrostatic repulsion at the surface of the anionic cell membrane. Therefore, effective delivery of siRNA to key targets is needed for clinical applications [16].

Poly (lactic-*co*-glycolic acid) (PLGA) can be used as a method for delivering siRNA to the target region. PLGA polymers have been found to be accomplished carriers for controlled administration of drugs, peptides, and proteins. Because of its biocompatibility and biodegradability, it has been approved for therapeutic applications by the U.S. Food and Drug Administration [17]. Particularly, PLGA has several characteristics suitable for siRNA delivery. First, PLGA particles are small enough to penetrate the tissue barrier and can be taken up by the cells. Second, they can entrap and release a sufficient amount of siRNA [18]. Third, the PLGA nanoparticle represents a promising tool that holds the potential to enhance microglial targeting by siRNAs due to its ability to protect siRNAs from inactivation and to improve its solubility and bioavailability [19].

NF-κB is bound and inhibited by the inhibitor of κB (IκB). Several factors such as growth factors, proinflammatory cytokines, chemotherapeutic agents, and antigen receptors activate the IκB kinase (IKK) complex, which phosphorylates IκB protein and results in ubiquitination and proteasome degradation resulting in the release of NF-κB. The released NF-κB is associated with neuropathic pain mediated through several pathways as mentioned above [20]. The IKK complex consists of three components: α, β, and γ. IKKα is involved in the noncanonical pathway, and IKKβ (also known as inhibitor of nuclear factor kappa B kinase subunit beta, IKBKB) is known to play an essential role in the canonical pathway [21]. Because NF-κB signaling plays a pathogenic role in a variety of inflammatory diseases, there are many therapeutic strategies for inflammatory diseases to block NF-κB activity. Accordingly, we aimed to specifically inhibit IKK by preventing phosphorylation of IKBKB for inflammatory disease and pain. In addition, it was recently reported that microglia activation decreased in model of nerve injury-induced neuropathic pain in IKKβ conditional knockout mice [22]. Nerve injury–induced microglia activation was greatly attenuated in *cIkkβ^−/−^* mice at 7 days after surgery. Therefore, it could be expected to reduce the neuropathic analgesic effect by reducing the expression of IKBKB using PLGA nanoparticles.

This study investigated the inhibition of microglial activation using *IKBKB* siRNA-encapsulated PLGA nanoparticles and investigated whether neuropathic pain is reduced in spinal nerve ligation (SNL) rat models, which showed spinal cord proliferation of microglial cells after nerve injury [23].

## 2. Results

### 2.1. Upregulation of Microglial Activation in Rats with Spinal Nerve Ligation-Induced Neuropathic Pain

**Figure 1:**
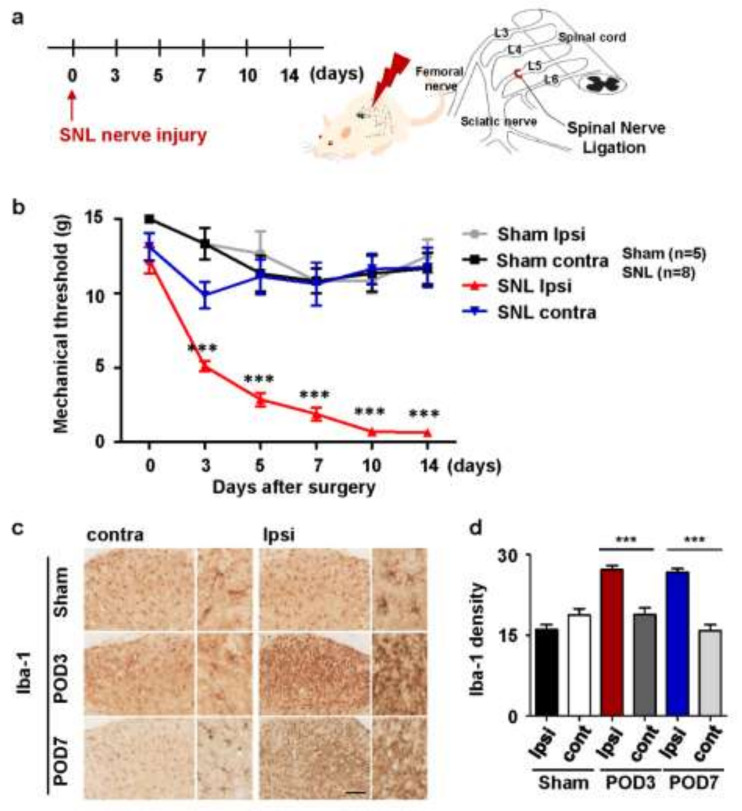
Microglial activation was induced by L5 spinal nerve ligation (SNL) in the rat neuropathic pain model. (**a**) Neuropathic pain was induced by ligation of L5. (**b**) Rats were then subjected to pain behavior testing using von Frey filaments for assessing the degree of neuropathic pain following SNL. Data are presented as mean ± standard error of the mean (SEM) (one-way analysis of variance with Tukey’s post hoc test, *** *p* < 0.001 versus ipsilateral of sham group, n = 8 per group). (**c**) On days 3 and 7 after SNL surgery, L5-level spinal cord sections were taken and immunostained with anti-Iba-1 antibodies. Scale bar = 100 µm. (**d**) Iba-1 immunostaining density was measured using Image J. Data are presented as mean ± SEM (one-way analysis of variance with Tukey’s post hoc test, *** *p* < 0.001 versus contralateral, n = 4 per group).

Before the experiment, microglial activation was confirmed in SNL rat models in which neuropathic pain was induced. Mechanical allodynia was assessed 3, 5, 7, 10, and 14 days after SNL surgery (Figure 1a). In all rats with SNL surgery, the mechanical threshold in the ipsilateral foot began to decrease on day 3, peaked on day 10, and continued until day 14. Rats that responded only to stimuli of 10 g or more showed avoidance responses to stimuli as low as 0.4 g. The sham group without L5 ligation did not show significant mechanical allodynia in the ipsilateral foot as did those without surgery (Figure 1b). Microglia were activated in the dorsal horn of the spinal cord ipsilateral to the affected area. To evaluate the activation of microglia, immunostaining was performed using anti-Iba-1 antibody 3 and 7 days after the surgery. In the SNL group, microglia were activated on both dates in the ipsilateral side compared to the contralateral side. In contrast, microglia in the sham group were not activated in either dorsal horn of the spinal cord (Figure 1c); however, the density increased by 1.5 to 2 times compared to the contralateral side in the sham group (Figure 1d).

### 2.2. Preparation and Characterization of IKBKB siRNA-Encapsulated PLGA Nanoparticles

**Figure 2:**
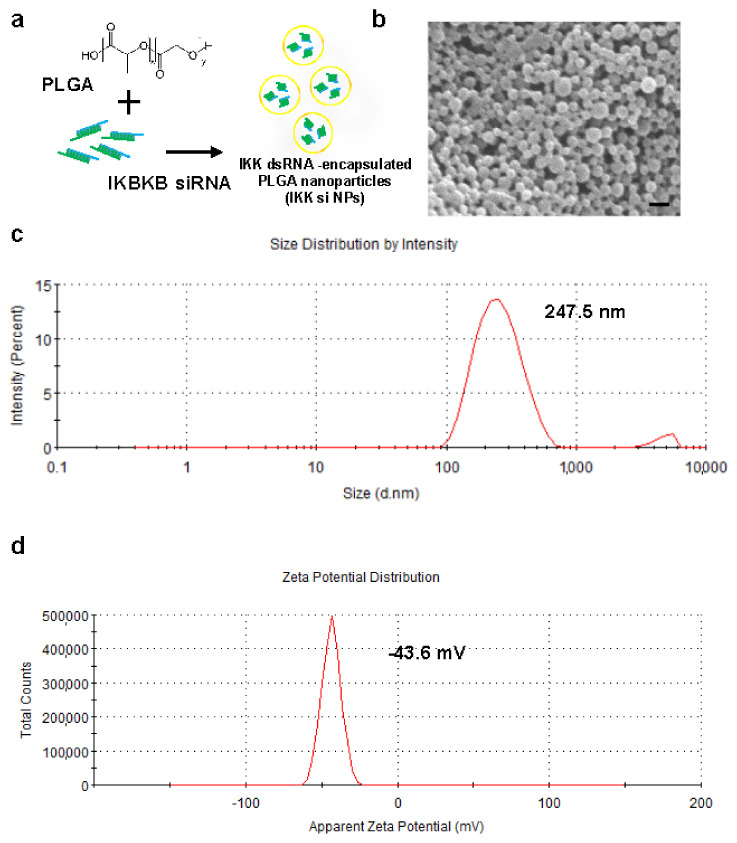
Physical characterization of the inhibitor of NF-κB kinase subunit beta (*IKBKB*) small interfering RNA (siRNA)-encapsulated poly (lactic-*co*-glycolic acid) (PLGA) nanoparticles. (**a**) *IKBKB* siRNA-encapsulated PLGA nanoparticles were prepared by sonicating a mixture of PLGA and *IKBKB* siRNA. (**b**) Suspended nanoparticles were assessed using scanning electron microscopy; scale bar = 200 nm. siRNA-encapsulated PLGA nanoparticles were dissolved in water and were measured for size (**c**) and zeta potential (**d**) using a Zetasizer ZS90.

There are several factors to consider while selecting a delivery system, such as stability, efficiency, convenience, and cost, for effectively delivering *IKBKB* siRNA to the spinal cord microglia. This is the process of synthesized nanoparticle for encapsulation of hydrophilic siRNA (Figure 2a). Moreover, the shape of the nanoparticles was confirmed using a scanning electron microscope (Figure 2b). The size and zeta potential of the siRNA-encapsulated PLGA nanoparticles measured using a Zetasizer were 247.5 nm and −43.6 mV, respectively (Figure 2c,d).

### 2.3. Cellular Uptake of Rhodamine Conjugated PLGA Nanoparticles and AAV-EF1α-mCherry Vector-Encapsulated PLGA Nanoparticles

Although the in vivo safety of PLGA nanoparticles has already been validated, it has not been tested on microglial cells. Moreover, as there are several types of cells in the spinal cord, including neurons, astrocytes, and microglia, these were all examined to be transfected with PLGA nanoparticles.

**Figure 3:**
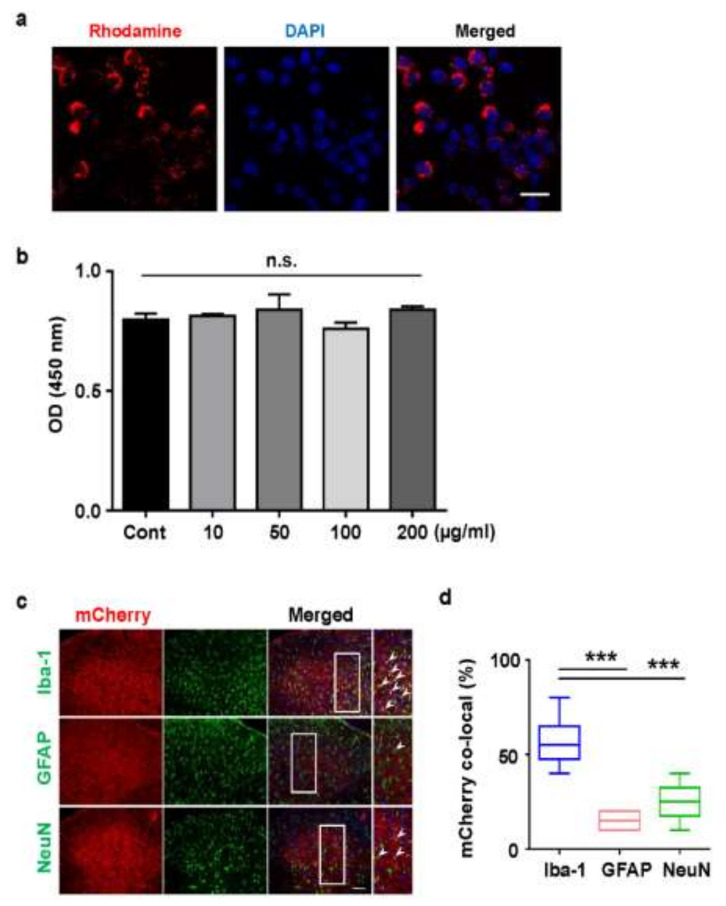
Cellular uptake and cytotoxicity of poly (lactic-*co*-glycolic acid) nanoparticles. (**a**) Rhodamine conjugated PLGA nanoparticle were incubated for 3 h at 50 µg/mL in BV2 cells and examined to assess cellular uptake. (**b**) BV2 cells were incubated with PLGA nanoparticles (0–200 µg/mL) for 24 h and examined for cytotoxicity by cell viability assay. Data are expressed as the mean ± SEM (one-way ANOVA test, not significant) (**c**) Three days after the intrathecal injection of AAV-EF1α-mCherry vector nanoparticles (20 μL), the L4–L6 section of the spinal cord was excised and was used for immunostaining with anti-Iba-1 (a microglial marker), anti-GFAP (an astrocytic marker), and anti-NeuN (a neuronal marker) antibodies for visualizing the distribution of AAV-EF1α-mCherry vector nanoparticles in the spinal cord. (**d**) The percentages of mCherry nanoparticle-incorporated microglia, astrocytes, and neurons in the dorsal horn of the spinal cord were counted. Five rats were used for each group, and three tissues were selected and stained for each sample. Data are presented as mean ± SEM (one-way ANOVA with Tukey’s post hoc test, *** *p* < 0.005 vs. Iba-1 positive mCherry co-localization %, n = 5 per group).

We investigated whether the microglia cell line, BV2 was uptake with PLGA nanoparticles. Because siRNA-included PLGA nanoparticles themselves do not fluoresce, Rhodamine conjugated PLGA nanoparticles emitting red fluorescence were prepared to trace their cellular uptake in real time. BV2 cells observed the endocytosis of Rhodamine conjugated PLGA nanoparticles (Figure 3a). Next, treatment of BV2 cells with IKBKB siRNA-loaded PLGA nanoparticles (0–200 μg/mL) had a minimal impact on cytotoxicity, as determined by MTT assay (Figure 3b), consistent with previous results (Figure 3b) [24]. Investigations were performed in vivo for assessing cell type-specific differences in PLGA nanoparticle uptake in the rat spinal cord. Since PLGA nanoparticles, and siRNA itself, do not fluoresce, AAV-EF1α-mCherry vector-loaded PLGA nanoparticles that emit mCherry red fluorescence were prepared for tracking cellular uptake. AAV-EF1α-mCherry vector-loaded PLGA nanoparticles were injected around the spinal cord through the intervertebral space using a Hamilton syringe. After three days, the nanoparticle-containing spinal tissue (L4-L6) was immunostained with anti-NeuN (neuronal marker), anti-GFAP (astrocyte marker), and anti-Iba-1 (microglia marker) antibodies (Figure 3c). Although AAV-EF1α-mCherry vector-encapsulated particles were observed in all cell types, 55% ± 5.57% co-localization of mCherry fluorescence was observed in microglia, 15% ± 2.23% was observed in astrocytes, and 25% ± 4.28% was observed in neurons. Ultimately, a two-times higher level of mCherry fluorescence was detected in microglia present in the dorsal horn of the spinal cord (Figure 3e). AAV-EF1α-mCherry vector encapsulated PLGA nanoparticles were non-toxic to all cells; it was taken up by all cell types present in the dorsal horn of the spinal cord, showing a higher uptake in microglia by phagocytosis.

### 2.4. Intrathecal Injection of IKBKB siRNA-Encapsulated PLGA Nanoparticles Diminishes Mechanical Hypersensitivity in SNL-Induced Rats

**Figure 4:**
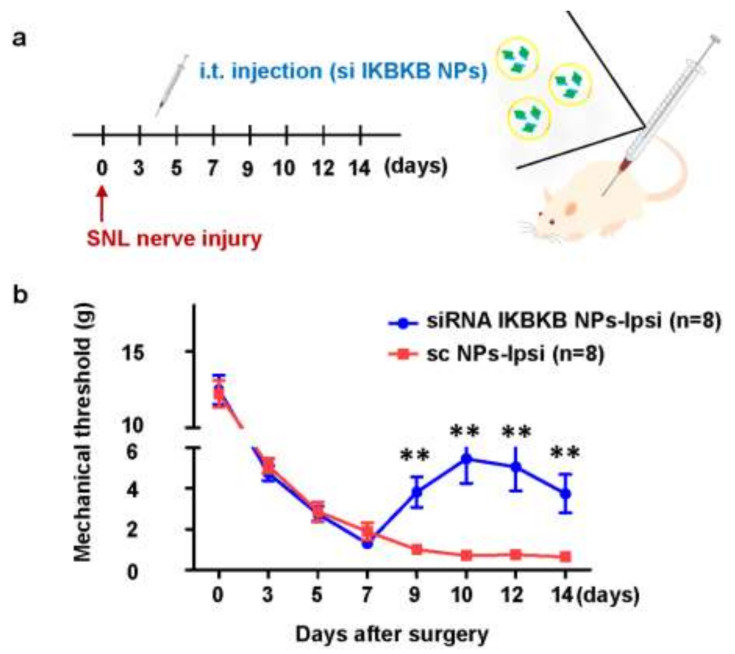
Intrathecal injection of PLGA-encapsulated *IKBKB* siRNA nanoparticles alleviates mechanical allodynia in rats with spinal nerve ligation. (**a**) Scramble siRNA or *IKBKB* siRNA nanoparticles were injected intrathecally at four days post-SNL nerve injury. (**b**) Rats were subjected to pain behavior testing using von Frey filaments for evaluating the effect of *IKBKB* siRNA on neuropathic pain. Data are presented as mean ± SEM (one-way ANOVA with Tukey’s post hoc test, ** *p* < 0.01 vs. sc NPs-Ipsi, n = 8 per group).

On the fourth day after the surgery, scrambled siRNA- and *IKBKB* siRNA-encapsulated PLGA nanoparticles were intrathecally injected through the intervertebral space with a Hamilton syringe (Figure 4a). To confirm mechanical pain, thresholds owing to mechanical stimulation were confirmed by von Frey filament testing at 3, 5, 7, 9, 10, 12, and 14 days after the surgery. In both the groups injected with *IKBKB* siRNA-encapsulated PLGA nanoparticles and scrambled siRNA-encapsulated PLGA nanoparticles, neuropathic pain increased until seven days after the surgery. When neuropathy was more severe, a withdrawal reaction, such as avoiding the sole of the foot was observed even with a stimulation of approximately 1 g. From seven days after SNL surgery, the group injected with PLGA nanoparticles encapsulated with *IKBKB* siRNA showed a pattern of reducing neuropathic pain (Figure 4b). The threshold for physical stimulation was highest on the tenth day after the surgery, i.e., on the sixth day after intrathecal injection of nanoparticles. During this time, the rats were observed to not respond to the stimulus of 5 g or less, after which it was observed that the threshold fell again.

In contrast, in the group injected with scrambled siRNA-encapsulated PLGA nanoparticles, the threshold decreased more and neuropathic pain increased. The most sensitive rat showed withdrawal behavior in response to 0.4 g of stimulation. These data show that intrathecal injection of *IKBKB* siRNA-encapsulated PLGA nanoparticles reduced SNL-induced neuropathic pain.

### 2.5. IKBKB siRNA-Encapsulated PLGA Nanoparticles Reduce Microglial Activation and Modulate the Expression of Neuropathic Pain-Related Genes in the Dorsal Horns of SNL-Induced Rat Spinal Cords

**Figure 5:**
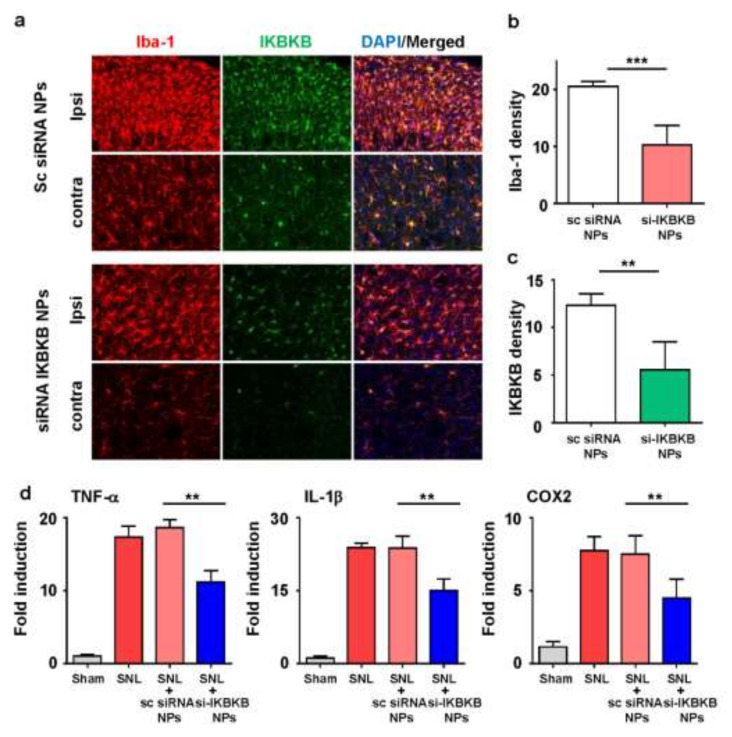
*IKBKB* siRNA-encapsulated PLGA nanoparticles attenuated the expression of proinflammatory mediators in SNL rat models. (**a**) On day six post-intrathecal injection, L5 spinal sections were taken and incubated with anti-Iba-1 and anti-*IKBKB* antibodies. Data are presented as mean ± SEM (*t*-test, ** *p* < 0.01 vs. sc siRNA NPs, n = 8 per group). Density of fluorescence intensity showing Iba-1 (**b**) and *IKBKB* (**c**). All data are presented as mean ± SEM of the three experiments. (**d**) On day 6 after injection, total mRNA was extracted from the dorsal horn of the ipsilateral spinal cord from L4–5 (0.7 cm) and used for cDNA synthesis. The mRNA levels of TNF-α, IL-1β, and cyclooxygenase (COX) 2 were measured using qRT-PCR. Data are presented as mean ± SEM (one-way ANOVA with Tukey’s post hoc test, ** *p* < 0.01 vs. sc siRNA NPs, n = 8 per group). sc, scramble; NPs, nanoparticles.

Considering the importance of NF-κB-mediated microglial activation in neuropathic pain, we hypothesized that *IKBKB* could be an important target for gene therapy in the treatment of neuropathic pain. We investigated whether *IKBKB* siRNA-encapsulated PLGA nanoparticles can attenuate microglial activity in the spinal cord of SNL-induced rats. As expected, the microglial activity in the ipsilateral dorsal horn four days after intrathecal injection was dramatically weakened in both the absolute number and cell density in the group injected with *IKBKB* siRNA-encapsulated PLGA nanoparticles compared to the control group (Figure 5a). Moreover, *IKBKB* siRNA nanoparticles reduced the activation of microglia in SNL rats, as indicated by lower Iba-1 immunoreactivity (Figure 5b). Staining of IgG was performed to check whether the IKBKB antibody selectively stains IKBKB protein (Supplementary Figure S1). Furthermore, the expression of *IKBKB* significantly decreased with the *IKBKB* siRNA-encapsulated nanoparticles compared with the control group (Figure 5c). This correlated with the results of von Frey filament tests indicating that the analgesic effects of *IKBKB* siRNA nanoparticle treatment peaked on postoperative day 10. The findings indicated that *IKBKB* siRNA nanoparticles mainly targeted microgliosis and microglial activity.

Following the confirmation of *IKBKB* expression, the release of microglial mediators was examined in the spinal dorsal horns of the rats using qPCR (Figure 5d). The mRNA expressions of TNF-α, IL-1β, and COX2 were evaluated in samples collected from the ipsilateral dorsal horn of the spinal cord on the sixth day after intrathecal injection. Based on the expression of the housekeeping gene GAPDH, each inflammatory factor was equilibrated and analyzed how many folds compared with control group. The mRNA levels of these proinflammatory genes significantly decreased in the group injected with *IKBKB* siRNA nanoparticles compared to the group injected with scrambled siRNA-encapsulated nanoparticles.

Altogether, *IKBKB* siRNA-encapsulated PLGA nanoparticles suppressed the expression of *IKBKB*, weakened the activity of NF-κB to reduce the activation of microglia, and thereby alleviated neuropathic pain in SNL-induced rats.

## 3. Discussion

Neuropathic pain is often chronic and difficult to treat. Pain relief is sometimes not sufficient even with drug therapy, such as NSAIDs, analgesics, antiepileptics, antidepressants, and opioids; there are cases in which the therapeutic effect is insufficient, and even if effective, a drug may have side effects and a short half-life [25]. Therefore, many studies have been conducted on new effective treatment methods for neuropathic pain. Microglia of the spinal cord are activated after peripheral nerve damage, and activation of microglia plays a causal role in the development of neuropathic pain. Based on these this concept, our research has placed importance on the mechanism of spinal cord microglia activation due to nerve damage.

In our study, we employed a method using siRNA-encapsulated nanoparticles targeting a specific gene to reduce microglial activation. It is known that microglial activation in the spinal cord plays an important role in the development of neuropathic pain in humans and rodents. Microglia are activated by neurotransmitters secreted from the terminals of presynaptic neurons in the dorsal horn of the spinal cord, and neuropathy is induced by the secretion of various inflammatory factors (TNF-α, IL-1β, and IL-6). Therefore, reducing microglial activation is expected to reduce neuropathic pain. Methods of directly targeting microglial cells, such as minocycline, have been tried, but there are problems with cytotoxicity, such as neurodegeneration. Research on the safe use of these drugs is ongoing, and their use is limited because of their short half-lives [25,26]. When neurotransmitters bind to the receptors on the microglial membrane, they cause migration, division, and other changes in the microglia, and various factors are involved in this process. NF-κB is one of the transcription factors that affect the development of neuropathic pain and whose activity is limited by IκB. When a specific signal stimulus is provided to IκB, IκB is phosphorylated and degraded, and free NF-κB enters the nucleus and is transcribed. IKK has several other proteins that phosphorylate IκB. There is alpha beta gamma as the type, and this experiment was conducted with beta known as a representative factor. Moreover, recently reported, deletion of IKBKB in DRG neurons reduced nerve damage-induced NF-κB stimulation in DRG and was associated with decreased upregulation of interleukin16 [27]. Targeting IKBKB could be more effective because many signaling pathways that active NF-κB converge at the IKBKB level.

It is crucial to examine how siRNA is delivered into the spinal cords of rats. Our previous study [24] introduced a novel nanomaterial-based approach for treating neuropathic pain. In addition, a previous study has demonstrated the reliable uptake of PLGA nanoparticles by microglia [28]. In this experiment, we went one step further and checked whether effective results were produced even when a factor that induces the expression of a specific gene was injected. Instead of injecting NF-κB directly, IKBKB, which induces NF-κB activity, was used. As a result, it was confirmed that the response to pain was significantly reduced in the group in which PLGA nanoparticles encapsulating IKBKB siRNA were injected into the intrathecal space. Moreover, it was confirmed that the activation of microglia was significantly reduced. In addition, the synthesis of pro-inflammatory mediators, such as TNF-α, IL-1β, and COX2 were reduced by IKBKB siRNA nanoparticles encapsulated in PLGA. These results suggest that silencing of the IKBKB gene inhibits the activity of NF-κB and, consequently, reduces the activation of microglia, thereby alleviating neuropathic pain. Therefore, through the delivery of specific siRNA-containing nanoparticles, not only can it be expected to effectively improve neuropathic pain, but it was also confirmed that it works as desired in the pathway over several steps. In this experiment, the mechanical threshold was lower than that of the control group until 10 days after injection of the nanoparticles. These effects may be due to the slow release of the drug from nanoparticles within the cell along with the therapeutic effects of siRNA. This is the part that can be expected to show pain relief for a longer duration compared to conventional drugs. In the future, research on the effects of siRNA-encapsulated nanoparticles on the body, side effects, long-term use, and repeated use is needed.

In summary, we demonstrated a novel target for spinal cord microglia activation after peripheral nerve injury via PLGA nanoparticle. Nerve injury-induced IKBKB induction leads to NF-κB upregulation in dorsal horn, which in turn induces spinal cord microglia activation and cytokine release. Targeting IKBKB expression by PLGA nanoparticle is a potential novel therapeutic strategy in the treatment of neuropathic pain.

## 4. Materials and Methods

### 4.1. Animals

Sprague–Dawley rats (six-week-old, male, 150–200 g) were purchased from Dae Han Bio Link (DBL, Chung-ju, Chung-buk, Korea) one week before the start of the experiment to give them sufficient time to adjust to the new environment. Three rats were placed per cage, with a 12-h night/day cycle and access to water and food at any time. This experiment was approved by Chungnam National University Animal Care and use committee (CNUH-019-A0040). Our experiment approved on 25 July 2019, and it was conducted from the date of approval until 31 May 2020. The ethical guidelines of the National Institutes of Health and International Association for the study of pain were followed [29].

### 4.2. Induction of Neuropathic Pain with L5 SNL

The rats used in this experiment had neuropathic pain induced by the ligation of the lumbar spinal nerve root 5 (L5), which is well established as a neuropathic pain model [25,30]. Alfaxan (Alfaxalone, 10 mg/cc, Jurox Animal Health, North Kansas, MO, USA) 8 mg was injected into the right thigh for anesthesia, and the rats were placed in the prone position. A 2-cm skin incision was created around the pelvis, and the left paraspinal muscles were removed. After removing the left transverse process of L6 to expose the L4 and L5 vertebrae, the L4 and L5 nerves were found and separated carefully to avoid injury. The L5 nerve was tied three times using a 3-0 silk thread (Ethicon, Diegem, Belgium). The wound was closed after confirming hemostasis. Identical surgery was performed in the sham group, except for the L5 nerve ligation.

### 4.3. Intrathecal Injection

Intrathecal injection through the lumbar intervertebral space was performed as previously described [26]. Briefly, rats were placed in the prone position on the operating table, and alfaxan was injected into the right thigh as a general anesthetic. Moreover, each 20 µL of siRNA-encapsulated 2.5 mg of PLGA NPs were incubated in 250 µL phosphate-buffered saline (PBS) solution. Next, 20 μL of nanoparticles in PBS solution, at pH 7.4, (*IKBKB* siRNA-encapsulated PLGA nanoparticles) was injected through the intervertebral space between L5 and L6 using a Hamilton syringe (100 μL; Reno, NV, USA) with a 26-G needle. Successful injections were confirmed by a clear tail-flick or cerebrospinal fluid regurgitation.

### 4.4. Pain Behavior Test with Von Frey Filaments

Pain behavior tests were assessed using von Frey filaments (NC12775-99, Touch Test^®^ Sensory Evaluators, 20-Piece Hand Kit, North Coast Medical & Rehabilitation Products, Morgan Hill, CA, USA) according to previous reports [28]. Continuous response was assessed with the up-down method using eight consecutive forces of von Frey filaments (force: 0.4, 0.6, 1, 2, 4, 6, 8, 15 g). The filament was placed vertically in contact with the left foot and was held for 5–6 s, and the response was considered positive if the rat exhibited a rapid avoidance or immediately jumped or licked the foot. Stimulation began with a weak filament. If there was no positive reaction, stimulation was performed with a stronger filament. The minimum stimulus size for a positive response was used as the threshold, and the upper limit for no response above 15 g was no longer applied.

### 4.5. Quantitative Reverse Transcription-Polymerase Chain Reaction 

Total RNA from the spinal cord was isolated using TRIzol reagent according to the manufacturer’s protocol (GeneAll, RoboEx^TM^, Thermo Fisher Scientific, Waltham, MA, USA). RNA was quantified using the NanoDrop spectrometer (Thermo Fisher Scientific, Waltham, MA, USA). cDNA was prepared from total RNA using a kit (Enzynomics, Daejeon, Korea). Quantitative polymerase chain reaction (qPCR) was performed using the AriaMax Real-time PCR system (Agilent Technologies, Santa Clara, CA, USA) with the TOPreal™ qPCR 2X premix (SYBR Green with low ROX; Enzynomics). The primers used for rat GAPDH, TNF-α, IL-1β, and COX2 were as follows: rGAPDH 5′-CTCATGACCACAGTCCATGC-3′, and antisense 5′-TTCAGCTCTGGGATGACCTT-CT-3′; rTNF-α 5′-AGATGTGGAACTGGCAGAGG-3′, and antisense 5′-CCCATTTGGGAACTTCTC-CT-3′; rIL-1β, 5′-CAGCAGCATCTCGACAAGAG-3′, and antisense 5′-CATCATC-CCACGAGTCACAG-3′; rCOX2 5′-CAGTATCAGAACCGCATTGCC-3′, and antisense 5′-GAGCAAGTCCGTGTTCAAGGA-3′.

### 4.6. PLGA Nanoparticles Preparation

PLGA nanoparticles with 50:50 ratio of lactic and glycolic acids carrying *IKBKB* siRNA (Thermo Fisher, Waltham, MA, USA) or scrambled siRNA (Thermo Fisher, Waltham, MA, USA) were prepared using an emulsification/solvent evaporation method [31]. To prepare PLGA nanoparticles, 200 µL (20 µM) of *IKBKB* siRNA or scrambled siRNA in diethyl pyrocarbonate water (Enzynomics) was added drop-by-drop to 800 µL dichloromethane (DCM, Samjeon, Korea) containing 25 mg PLGA (Corbion, Amsterdam, The Netherlands) and was emulsified using sonication (Branson Digital Sonifier, SFX 550, EMERSON, Danbury, CT, USA) into a primary W1/O emulsion. Then, 2 mL of 1% PVA1500 (*w/v*) was added directly to the primary emulsion and further emulsified by sonication to form a W1/O/W2 double emulsion. The resulting product was diluted with 6 mL of 1% PVA1500 and magnetically stirred at room temperature (20–22 °C) for 3 h to evaporate DCM. The resulting PLGA nanoparticles were collected using ultracentrifugation at 13,000 rpm for 10 min at 4 °C. and they were washed once with deionized RNase-free water, resuspended in water, and finally lyophilized. Physical characteristics of the nanoparticles were analyzed with the Zetasizer Nano ZS90 (Malvern Instruments, Enigma Business Park, Grovewood Road, Malvern, UK) and scanning electron microscopy (SNE-3500, SEC, Korea) [32].

### 4.7. Histological Analysis and Immunohistochemistry

Rats were anesthetized and perfused with PBS (pH 7.4) followed by 4% paraformaldehyde (Merck, Darmstadt, Germany) in PBS using a peristaltic pump at 20 mL/min. The lumbar region (L4–L6) of the spinal cord was obtained and immunohistochemical analysis was performed. Spinal cords were sectioned coronally at 35 µm using a freezing microtome, and sections were stored in a storage buffer. Nuclei were counterstained with Hoechst 33342 and visualized with a confocal microscope (Leica, TCS SP8, Wetzlar, Germany). The sections were incubated with primary anti-Iba-1 (1:200; Abcam, Cambridge, UK), anti-GFAP (1:200; Millipore, Burlington, VT, USA), anti-NeuN (1:200; Cell Signaling, Danvers, MA, USA) and anti-IKK beta (1:200; Abcam, Cambridge, UK) antibodies overnight. Immunostaining was performed using the avidin-biotin peroxidase complex (ABC) method as reported previously [33]. Immune densities in the graphs were quantified using Image J software (National Institutes of Health, Bethesda, MD, USA). 

### 4.8. Statistical Analyses

Data are expressed as means ± standard errors of the mean (SEM). Differences between the two groups were evaluated using the *t*-test. One-way analysis of variance was performed for multiple comparisons with the Dunnett’s post hoc test. All statistical analyses were performed using GraphPad Prism 6 (GraphPad Software, San Diego, CA, USA). All data are representative of at least three independent experiments. A *p*-value of < 0.01 was considered statistically significant.

## Data Availability

Not applicable.

## Supplementary Materials

The following are available online at https://www.mdpi.com/article/10.3390/ijms22115657/s1, Figure S1: Immunofluorescence assay of anti-Rabbit IgG.
