# Supplementary figures and images for: IKBKB siRNA-Encapsulated Poly (Lactic-co-Glycolic Acid) Nanoparticles Diminish Neuropathic Pain by Inhibiting Microglial Activation
Source: Int J Mol Sci. 2021 May 26;22(11):5657. doi: 10.3390/ijms22115657 (PMC8203094; doi:10.3390/ijms22115657)

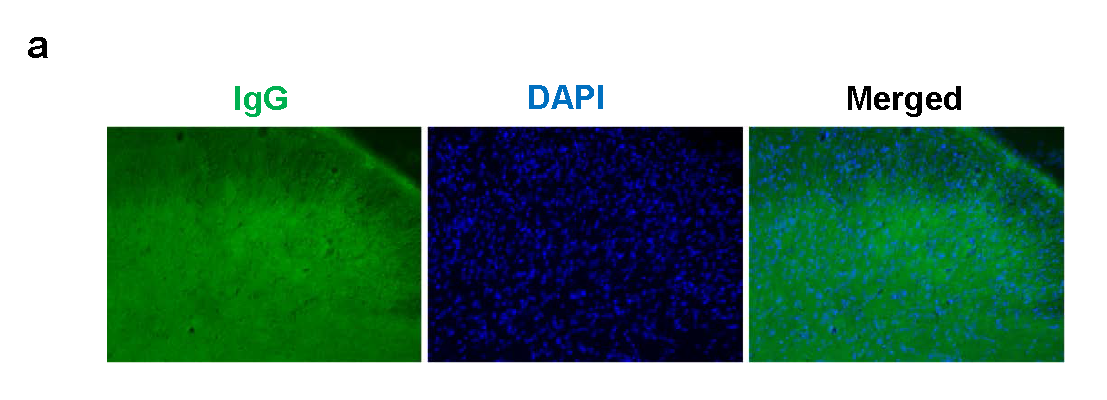

Supplement: Supplementary file 1 [file ijms-22-05657-s001.zip › ijms-1239193-supplementary.tiff]
